# Structure–Function Relationship of Retinal Ganglion Cells in Multiple Sclerosis

**DOI:** 10.3390/ijms22073419

**Published:** 2021-03-26

**Authors:** Khaldoon O. Al-Nosairy, Marc Horbrügger, Sven Schippling, Markus Wagner, Aiden Haghikia, Marc Pawlitzki, Michael B. Hoffmann

**Affiliations:** 1 Department of Ophthalmology, University Hospital Magdeburg, 39120 Magdeburg, Germany; khaldoon.alnosairy@med.ovgu.de (K.O.A.-N.); mwagner87.mail@googlemail.com (M.W.); 2 Department of Dermatology, University Hospital Magdeburg, 39120 Magdeburg, Germany; marc.horbruegger@med.ovgu.de; 3 Multimodal Imaging in Neuro-Immunological Diseases (MINDS), University of Zurich, 8057 Zurich, Switzerland; s.schippling@gmx.de; 4 Center for Neuroscience Zurich (ZNZ), ETH Zurich, 8057 Zurich, Switzerland; 5 Department of Neurology, University Hospital Magdeburg, 39120 Magdeburg, Germany; aiden.haghikia@med.ovgu.de; 6 Department of Neurology, Institute of Translational Neurology, University Hospital Münster, 48149 Münster, Germany; Marc.Pawlitzki@ukmuenster.de; 7 Center for Behavioral Brain Sciences, 39120 Magdeburg, Germany

**Keywords:** retinal ganglion cells, optical coherence tomography, multifocal pattern electroretinogram, multiple sclerosis, ganglion cell layer, optic neuritis, peripapillary retinal nerve fiber layer, outer retinal layers

## Abstract

The retinal ganglion cells (RGC) may be considered an easily accessible pathophysiological site of degenerative processes in neurological diseases, such as the RGC damage detectable in multiple sclerosis (MS) patients with (HON) and without a history of optic neuritis (NON). We aimed to assess and interrelate RGC functional and structural damage in different retinal layers and retinal sites. We included 12 NON patients, 11 HON patients and 14 healthy controls for cross-sectional multifocal pattern electroretinography (mfPERG) and optical coherence tomography (OCT) measurements. Amplitude and peak times of the mfPERG were assessed. Macula and disc OCT scans were acquired to determine macular retinal layer and peripapillary retinal nerve fiber layer (pRNFL) thickness. In both HON and NON patients the foveal N2 amplitude of the mfPERG was reduced compared to controls. The parafoveal P1 peak time was significantly reduced in HON only. For OCT, parafoveal (pfGCL) and perifoveal (pGCL) ganglion cell layer thicknesses were decreased in HON vs. controls, while pRNFL in the papillomacular bundle sector (PMB) showed reductions in both NON and HON. As the mfPERG derived N2 originates from RGC axons, these findings suggest foveal axonal dysfunction not only in HON, but also in NON patients.

## 1. Introduction

The retinal ganglion cells (RGC) are of unrivaled interest not only in the investigation of the ophthalmological conditions, but also in many inflammatory and neurodegenerative diseases of the central nervous system (CNS). In fact, the reliable in vivo assessment of the RGC using optical coherence tomography (OCT) has generated evidence suggestive of the RGC loss alongside with the optic nerve forming axons in disorders such as Parkinson disease (PD) [1] and multiple sclerosis (MS) [2]. MS is a CNS autoimmune disease, characterized by inflammatory demyelination and neurodegeneration. The near to ubiquitous involvement of the visual system in MS [3] and the presence of unmyelinated retinal nerve fibers, which are directly accessible render the retina an ideal model to interrogate disease associated inflammatory and degenerative processes.

In MS cases with a history of optic neuritis (HON), RGC injury along with the RGC axons at the optic disc, i.e., the peripapillary retinal nerve fiber layer (pRNFL), is well established [2]. Further, recent evidence unveiled structural damage to RGC and pRNFL even in MS patients without a history of ON (NON) [4,5]. Outer retinal layers have also gained interest in the assessment of MS damage. Saidha et al. [6] reported thinning of the outer and inner nuclear layers in a subset of MS patients that might be due to primary retinal changes in MS. Wicki et al. reviewing the OCT utility in MS [7] reported that the ganglion cell inner plexiform layer (GCIPL) and the inner nuclear layer (INL) are the most widely studied in MS with potential biomarker properties. Further, they indicate that OCT might also provide hallmarks of the posterior visual pathway in MS, where pRNFL thinning in NON might be induced by trans-synaptic retrograde degeneration (via the lateral geniculate nucleus). It was demonstrated that 35–40% of pRNFL loss in NON is associated with posterior visual pathway pathologies [7], i.e., lesions within the optic radiation [8].

Electroretinography (ERG) measures of retinal function might corroborate and elucidate structural alterations of the retina in MS patients. The transient pattern ERG (PERG) N95 amplitude is an RGC driven response [9,10] and found to be reduced in affected optic nerves, as in HON [9,11,12]. Another ERG-derived parameter indicative of RGC (dys)function is the photopic negative response of the full field ERG (PhNR_ffERG_) [13]. Previous studies reported a reduced PhNR_ffERG_ in HON and NON [14]. Further, the focal macular PhNR (PhNR_focal_) confirmed RGC alterations in HON [15]. It remains unclear though whether MS damage alters RGC function in NON/HON coinciding with or preceding structural damage and whether that applies and extends to retinal layers beyond the RGC.

Only few studies reported outer retinal layers to be functionally altered consistent with [6] or even prior to structural changes in NON [16,17], although others did not detect either changes [18]. One study employing multifocal visual evoked potentials (mfVEP) reported reduced amplitudes and delayed peak time for both HON and NON associated with structural changes at the GCIPL and pRNFL [19]. Another study measuring ffERG, pattern VEP and mfERG in patients with subclinical MS observed abnormal cone responses in the ffERG, delayed VEP-P100, but no alterations of the mfERG [20]. Further, Nakamura et al. [15] showed that HON related changes in outer and inner retinal layers were related to the onset of ON, where a- and b-wave amplitudes of PhNR_focal_ recovered after 6 months of the ON onset in contrast to persistent reduction of the PhNR amplitude. On the other hand, Wilkins et al. [21] used full field electroretinography (ffERGs) to demonstrate that subclinical rod and cone dysfunction were associated with structural deficits of the RGC in NON, which suggests an abnormality of both outer and inner retinal layers. Sriram et al. [4] showed in NON delayed b-wave peak time of the PhNR_ffERG_, i.e., dysfunction outer to RGC, together with the presence of changes at the pRNFL and GCL, i.e., structural changes at RGC.

Multifocal pattern electroretinogram (mfPERG) is another technique which allows for a topographical analysis of retinal changes, where its P1 and N2 components are direct functional measures of the RGC the bodies and axons, respectively [10]. Therefore, P1 and N2 waves might serve as a functional biomarker for optic nerve damage. Accordingly, we hypothesized that the P1 and N2 waveforms might reveal RGC changes in NON prior or coincident with structural retinal damage. Thus, the present study aims are two-fold: i) To assess mfPERG changes in MS patients vs. controls and ii) To investigate the interrelationship of functional-structural measures at different retinal sites and layers. Here, we report the mfPERG-N2 to be reduced in both NON and HON along with peripapillary retinal fiber layer loss at the papillomacular bundle sector. Further, P1 reduced peak time suggests a retinal inflammatory process in the HON. Outer retinal layers’ structure and function was intact and comparable between all groups. Finally, we found an association between structural and functional measures only for central RGC axon dysfunction (mfPERG-N2) and perifoveal GCL.

## 2. Results

14 healthy controls, 12 NON and 11 HON participants underwent mfPERG recordings and OCT scans. Demographic and clinical characteristics of the participants are given in Table 1. Only one eye was randomly selected for the analysis if both eyes were eligible-see methods.

### 2.1. Functional Changes in NON and HON

**Figure 1:**
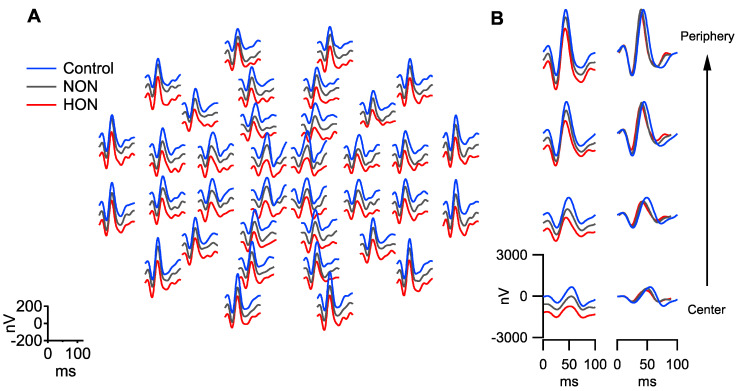
Grand mean traces of each group. (**A**) The grand mean of each of the 36 elements of the mfPERG stimulus. (**B**) The summed trace for the 4 rings of the mfPERG stimulus from the center to the periphery, i.e., central ring (ring 1): 4 elements; ring 2: 8 elements; ring 3: 12 elements; ring 4: 12 elements. Traces are offset between groups for A and left panel in B for clarity.

For a qualitative overview the grand mean trace arrays for the three participant groups are given in Figure 1A. To assess eccentricity dependent effects, the mfPERG responses were averaged within each eccentricity (“ring”) for each individual; the corresponding grand means of these ring averages are depicted in Figure 1B. For a quantitative assessment, the mfPERG components were identified for each individual and ANOVA or Kruskal–Wallis test (see Methods) were conducted to test the differences between groups of both amplitudes and peak times within each ring and across all rings Table 2.

In each individual, the 36 elements were averaged representing the global response of the mfPERG. For both amplitudes and peak times of the global response there were no significant differences between either MS groups and the healthy controls. mfPERG N1 (F(2,34) = 1.2, *p* = 0.3) and P1 (F(2,34) = 0.8, *p* = 0.5) summed amplitudes showed no significant effects in HON or NON vs. healthy controls. No significant N1 or P1 peak time effects were evident (H(2) = 4.9, *p* = 0.16 and H(2) = 5.0, *p* = 0.24, respectively). No group differences of the inner retinal response, i.e., N2 amplitude (F(2,34) = 1.2, *p* = 0.3) and peak time (H(2) = 0.1, *p* = 0.96) were observed.

**Figure 2:**
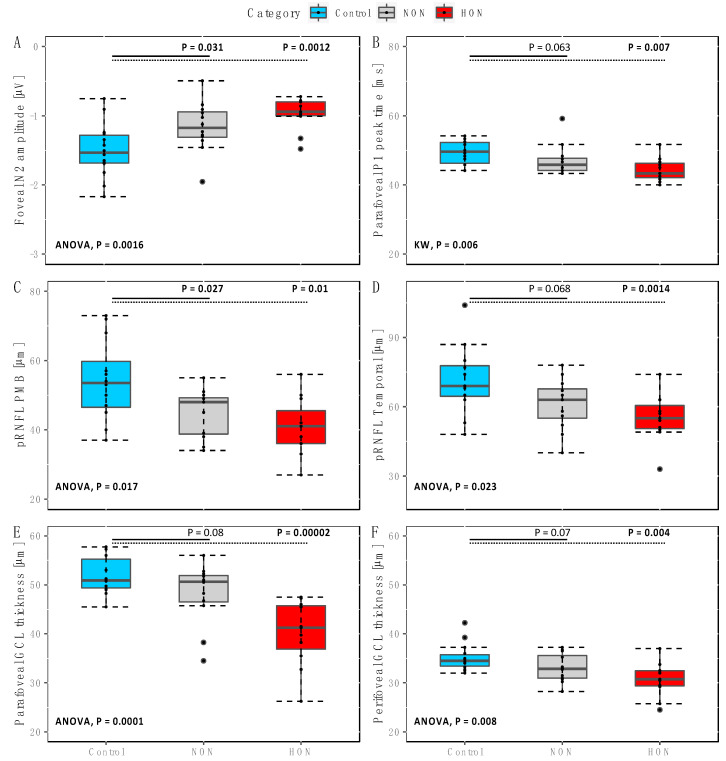
Post hoc analysis of ERG and OCT parameters. (**A**) Comparison of foveal (ring 1) N2 amplitude between groups with P values comparisons between MS without optic neuritis (NON) vs. controls (solid line) and MS with a history of optic neuritis (HON) vs. controls (dotted line). (**B**) Parafoveal P2 peak time (ring 2) significantly different between controls and HON. (**C**) Peripapillary retinal nerve fiber layer thickness at the papillomacular bundle (pRNFL PMB) also with significant differences between either patient groups vs. controls. (**D**) pRNFL of the temporal sector with only significant difference between HON and controls. (**E**,**F**) Parafoveal and perifoveal ganglion cell layer (GCL) with significant difference between HON and controls. Boxplots: lower whisker = smallest observation ≥ lower hinge—1.5 * Interquartile range (IQR); lower hinge: 25% quantile; median; upper hinge: 75% quantile; upper whisker = largest observation ≤ to upper hinge + 1.5 * IQR. Significant differences highlighted in bold.

For the assessment of individual eccentricities, only the amplitude of central ring N2 was significantly reduced in MS patients compared to controls (F(2,34) = 7.8, *p* = 0.0016). A post hoc analysis (Figure 2) revealed significantly lower N2 amplitudes of the central ring in NON (mean ± SD (µV): −1.16 ± 0.36) and HON (−0.97± 0.24) compared with controls(−1.5 ± 0.39, *p* = 0.031; *p* = 0.0012, respectively).

Other indicators of amplitude responses i.e., P1 and N1, did for neither eccentricity show significant differences between groups. Likewise, peak times within each ring for the respective mfPERG peaks did not show any delays or reductions in the patient groups. The P1 peak time in ring 2 (parafoveal), however, showed significant differences between groups (H(2) = 10.2, *p* = 0.024), where a post hoc analysis revealed significantly reduced peak times in the HON group vs. the healthy controls (median and range (ms): 43.33 and 11.67 vs. 49.58 and 10.0, *p* = 0.007).

### 2.2. Structural Changes in NON and HON

For structural changes at the peripapillary retinal area (Table 3), there were significant differences of the pRNFL thickness (µm) between groups at the papillomacular bundle (pRNFL PMB) (F(2,32) = 6.5, *p* = 0.017) and the temporal (pRNFL T) sectors (F(2,32) = 5.2, *p* = 0.023). A post hoc analysis revealed that eyes with NON (mean ± SD (µm): 45.00 ± 06.81 vs. 54.33 ± 11.77, *p* = 0.027) and HON (40.82 ± 08.30 vs. 54.33 ± 11.77, *p* = 0.01) showed lower mean thicknesses of the pRNFL PMB compared with controls.

At the macula, the analysis of the differences within ETDRS rings showed an absence of group differences for the thickness for all measures taken for the central 1 mm ring (Table 3).

At the parafoveal and perifoveal areas of the ETDRS scans, significant thickness reductions in MS groups were only observed for the pfIPL, pfGCIPL and pGCIPL (F(2,34) = 8.2, 13.8 and 6.5, *p* = 0.004, 0.0001 and 0.008, respectively). At the parafovea (Figure 2), the pfGCL thickness was significantly lower in the HON vs. controls (mean ± SD (µm): 40.14 ± 6.7 vs. 51.75 ± 3.7, *p* = 0.00002). Likewise, at the perifovea the pGCL thickness was significantly reduced in HON compared to controls (mean ± SD (µm): 30.73 ± 3.5 vs. 35.16 ± 2.8, *p* = 0.004).

### 2.3. Structural-Functional Correlation

**Figure 3:**
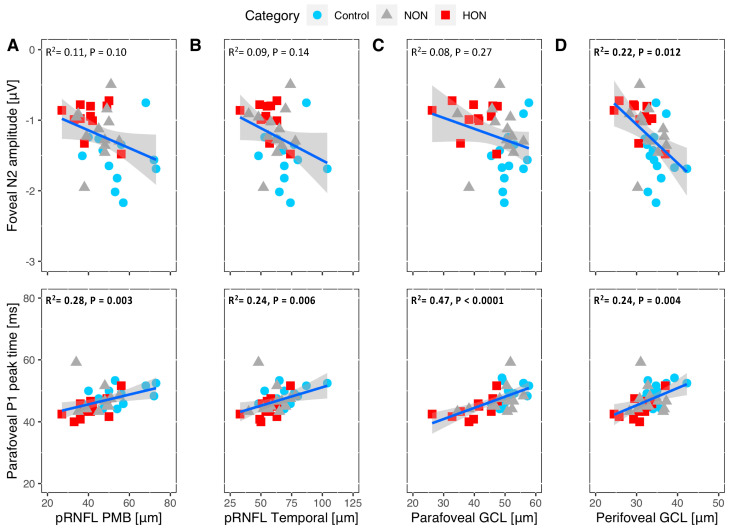
Association of foveal (ring1) N2 amplitude of the mfPERG reflecting RGC axon integrity (top row) and of the parafoveal (ring 2) P1 peak time of the mfPERG reflecting RGC body integrity (bottom row) vs. relevant structural tests. The *y*-axis is N2 (ring1) amplitude in the top row and P1 (ring2) peak time in the bottom row. A and B x axes: peripapillary retinal layer thickness at the papillomacular bundle sector (pRNFL PBM) and temporal sector (pRNFL T). C & D x axes: parafoveal and perifoveal ganglion cell layer thickness (GCL). Significant associations highlighted in bold.

Based on the comparative analysis, the correlation between functional and structural measures were assessed for the above reduced indexes in the MS subgroups, i.e., ring1 (foveal) N2 amplitude, pRNFL papillomacular bundle (PMB) and pRNFL temporal sectors (T) thickness and parafoveal and perifoveal GCL. The RGC central ring amplitude, i.e., N2, correlated significantly only with the perifoveal GCL thickness (Figure 3 top row; R^2^ = 0.22, *p* = 0.012). As an exploratory overview, we also report on the correlations for all mfPERG rings vs. the relevant structural measures (Table S1).

Moreover, P1 peak time of ring 2 (parafoveal) showed significant positive correlation with the significantly different structural measures between groups (Figure 3 bottom row). The highest P1 peak time association was with the parafoveal GCL thickness (R^2^ = 0.28, *p* < 0.0001).

## 3. Discussion

### 3.1. RGC Readout Using mfPERG-N2 vs. RGC and pRNFL Thickness

*Findings in HON.* Electrophysiological measures of retinal function allow for an objective evaluation of RGCs, a potential site of structural damage in MS. The N2 amplitude of the transient PERG or mfPERG reflects RGC axonal function [10,22], while OCT enables structural quantifications of both inner and outer retinal layers. Our findings in HON of reduced N2 amplitude of the mfPERG are in accordance with previous reports on RGC dysfunction in HON, notwithstanding their application of different methods to tap RGCs, i.e., conventional PERG [12,18] and PhNR_ffERG_ [14], or to tap the axon of the RGCs along the optic nerve downstream to the visual cortex, i.e., with mfVEP [19]. These previous studies also demonstrated structural changes, reduced pRNFL or GCIPL/GCL thickness [18,19], and significant structure function correlations [14,19]. The changes in HON reported in the present and in previous studies might be related to retrograde degeneration of optic nerve axons and RGC bodies following a demyelinating event at the level of the optic nerve or even posterior to it [14]. In accordance, previous combined diffusion magnetic resonance imaging (MRI) and OCT studies reported retrograde, i.e., GCIPL/pRNFL, as well as anterograde, i.e., optic radiation, transsynaptic degeneration [23] and associated brain volume reduction [24].

*Findings in NON.* There are only few previous studies directly assessing RGC structure and function in NON and these reported heterogenous findings. One study [14] applied PhNR_ffERG_ and found reduced PhNR amplitudes in NON in the absence of structural damage in pRNFL, the other [18] reported an absence of PERG albeit structural damage in NON. We confirm the former study by demonstrating a reduced N2 amplitude of the mfPERG and add evidence of structural damage in NON. Our report is also in accordance with visual function assessment in NON at a higher level, i.e., the visual cortex. Specifically, studies employing mfVEP reported optic nerve dysfunction in NON [25], diminished thickness of GCIPL/GCL and pRNFL [4,19] and significant correlations between OCT, mfVEP [4,19] and also optic radiation lesions, i.e., a higher degree of demyelination in the entire visual pathway [19]. This was further supported by the correlation of pRNFL and MRI changes at the optic radiation demonstrating transsynaptic retrograde degeneration [8,26]. Our study confirms the above findings of structural retinal abnormalities in NON and extends these findings to abnormalities in retinal function.

It should be noted that for both HON and NON vs. Controls the present study did not report significant differences the axon-layer of RGC at the macula, i.e., the macular retinal nerve fiber layer (mRNFL). In NON, few studies [17,27] reported in line with our findings no significant mRNFL alterations in contrast to consistent findings of decreased mRNFL in HON-see review [28]. We, however, believe that the most robust biomarker of RGC damage in MS lies within macular GCIPL and peripapillary RNFL as a recent review [28] concluded.

### 3.2. RGC Readout Using mfPERG-P1 vs. RGC and pRNFL Thickness

The reduced peak time in HON demonstrated in our study might be due to several factors. It is established that P1 [10] largely arises from the RGC body, while the N2 appears to be associated with the RGC axons themselves. Porciatti and Ventura [29] proposed that different onset response timings are due to the mixed presence of different RGC types for different stimulation conditions, i.e., magnocellular RGC and parvocellular RGC. Few studies of diseases that affect the RGC, i.e., glaucoma, found a shortening of the PERG P50 peak time or the steady state PERG phase, in established or suspect glaucoma patients with or without amplitude reduction [30,31]. One study [30] suggested that Porciatti and Ventura’s proposition [29] of early loss of RGC subgroups in glaucoma is a reason for this shortening. Comparable to our findings, significant P50 peak time or steady states PERG phase effects were positively correlated with structural measures, i.e., macular, pRNFL, and macular RNFL thickness [30].

Another possible explanation of the peak time shortening might arise during inflammatory retinal conditions causing excitatory ERG abnormalities [17]. Hood et al. proposed decreased peak times in conditions that lead to hyperexcitability of the bipolar-on cell and damage to the bipolar-off cells in monkey studies [32]. Ikeda et al. [33] demonstrated supernormal ERG timings in the early inflammatory ocular diseases, as well. Filgueiras et al. [17] supported the presence of retinal inflammatory changes in HON patients by reporting reduced peak times of the mfERG N1 and P1 waves. Despite the aforementioned evidence, further studies with larger sample size should explore reduced peak time in MS.

### 3.3. Outer Retinal Layers

In the present study, we found neither structural alterations in inner/outer nuclear layers (INL/ONL) and outer retinal layers (ORL) nor functional changes, i.e., N1. This might be indicative of the absence of the pathologies in these outer retinal layers, i.e., afferents of the RGC. It must be noted, however, that previous studies did report outer retinal involvement in MS, albeit in conflicting observations. Using mfERG, which reflects retinal bipolar and photoreceptor function, Hanson and colleagues [16] found delayed P1 peak time in MS independent of ON events, but in the absence of corresponding structural changes. In contrast, Filgueiras et al. [17] reported reduced peak times of the N1 and P1 of the mfERG, attributed to an inflammatory condition in the retina of HON. Saidha and colleagues [6] also supported the presence of primary retinal pathology by reporting disproportionate thinning of the inner and outer nuclear layers in OCT analysis in a subset of NON patients with reduced P1 amplitude of the mfERG. Gundogan et al. [20], on the other hand, reported no mfERG alterations, but ffERG-related outer retinal dysfunction. Different methods used, small sample size and cross-sectional nature of the studies are presumed reasons for these heterogenous findings.

### 3.4. Structure and Function Relationship of the RGC Measures

In our study, we found a significant correlation between central N2 amplitude and perifoveal GCL that was in part in agreement with another study reporting a significant correlation of transient PERG N95 with GCL thickness and pRNFL [18]. The reduced parafoveal mfPERG-P1 peak time, on the other hand, correlated significantly with parafoveal/perifoveal GCL and T/PMB pRNFL thickness. As a consequence, combining both diagnostic methods might support the detection of RGC loss along with axonal loss in MS.

In conclusion, the present study provides retinal, electrophysiological evidence of RGC axonal damage in both NON and HON, possibly as part of retrograde transsynaptic degeneration. Further, in HON reduced mfPERG P1 peak times suggest RGC abnormalities that might be associated with what is believed to be a retinal inflammatory process causing hyperstimulation of RGC bodies. Further, combined PERG and OCT assessment might provide a paradigm to integrate structure–function domains of visual function testing for enhancement of care in MS. A longitudinal study following up NON in a greater sample remains an area of great interest. Further, indications of a primary retinal pathology motivate further exploration and supplementation with additional diagnostic tests.

## 4. Materials and Methods

### 4.1. Participants

This study followed the tenets of the declaration of Helsinki and the protocol was approved by the ethical committee of the Otto-von-Guericke University of Magdeburg, Germany (No 74/14, 2014). This prospective observational study was conducted at the departments of Ophthalmology and Neurology. An informed written consent was obtained from all participants.

*MS.* Twenty three patients with a confirmed diagnosis of clinically definite relapsing-remitting MS according to the 2010 McDonald criteria [34] were enrolled in this study. Definition of the MS with ON (HON): Patients with a single history of optic neuritis at least one year ago. While definition of the MS without optic neuritis (NON): Patient without evidence of clinical or subclinical (normal visual evoked potential (VEP) peak time) ON. VEP data were published in another study [23]. The Expanded Disability Status Scale (EDSS) was used for the quantification of clinical disability, where lower values indicate less disability [35].

*Controls.* Fourteen healthy subjects with normal visual acuity (best corrected visual acuity (BCVA) ≤ logMAR of 0.00) participated in the study.

All participant groups underwent complete ophthalmic examinations, best corrected visual acuity testing (Early Treatment for Diabetic Retinopathy Study’s chart (ETDRS chart)), and visual field testing (Standard automated perimetry (dG2; dynamic strategy; Goldmann stimulus size III; OCTOPUS^®^ Perimeter 101, Haag-Streit International, Switzerland)). Exclusion criteria were any other systemic and/or ophthalmic diseases and refractive error exceeding ±5 D. One eye was randomly selected in the analysis, if both eyes of the participant were eligible.

### 4.2. mfPERG stimuli, Procedure, Recordings and Analysis

**Figure 4:**
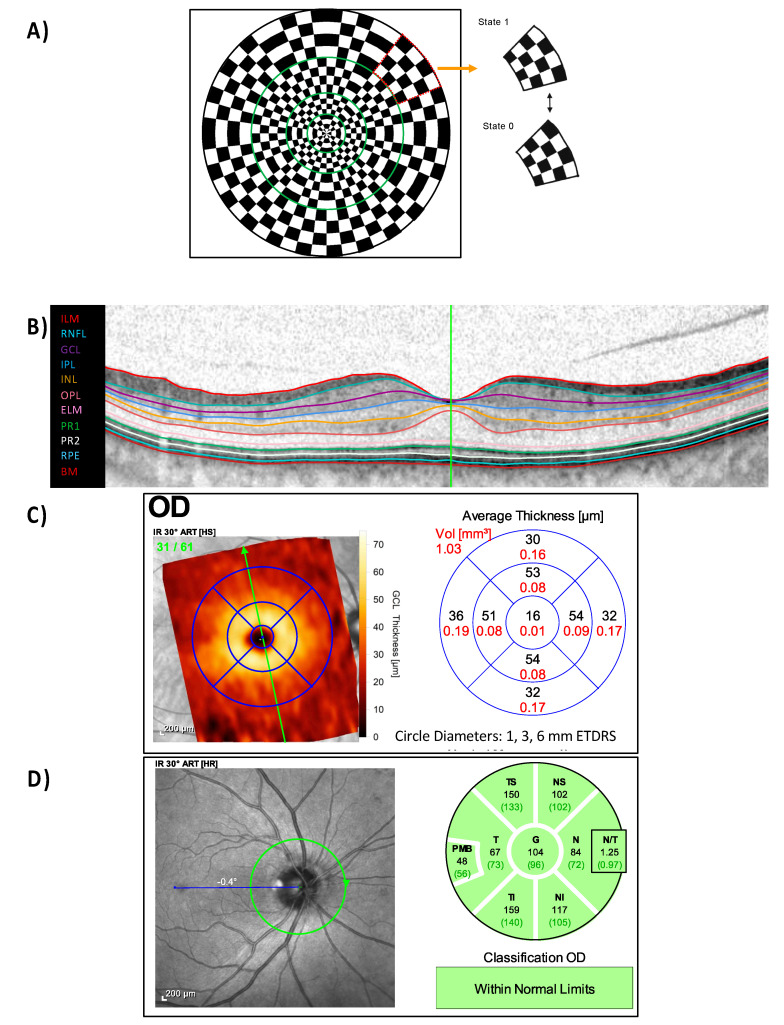
Electroretinography (ERG) and optical coherence tomography (OCT) measurements. (**A**) mfPERG stimulus with 36 checkerboard elements, where each element (dotted red) reverses the pattern between 1 and 0 states. The stimulus (xx° diameter) spanned 4 eccentricity ranges outlined by green line. (**B**) Macular vertical scan with segmentation line, (**C**) Early Treatment for Diabetic Retinopathy Study’s (ETDRS) circle, i.e., 1 mm (foveal), 3 mm (parafoveal), and 6 mm (perifoveal) circles, analysis of the ganglion cell layer (GCL), and (**D**) peripapillary area scan with different sector thickness. ILM = internal limiting membrane; RNFL = retinal nerve fiber layer thickness; GCL = ganglion cell layer; IPL = inner plexiform layer; INL = inner nuclear layer; OPL = outer plexiform layer; ELM = external limiting membrane; PR1 = photoreceptor inner segments; PR2 = photoreceptors outer segments; RPE = retinal pigment epithelium; BM = Bruch’s membrane. Peripapillary retinal nerve fiber layer thickness: G = global average thickness; T = temporal; PMB = papillomacular bundle; N/T: nasal/temporal ratio; S: superior; I: Inferior.

For stimulus delivery and electrophysiological recordings, we used VERIS 5.1.12XScience (EDI: Electro-Diagnostic Imaging, Redwood City, CA, USA). The stimulus covered 44° of visual field and comprised of 36 elements within 4 rings spanning the following eccentricities (Figure 4): 0.0–3.6, 3.6–7.6, 7.6–14.3 and 14.3–22.7°. For each element in the stimulus, the 4 × 4 checkerboard was stimulated with a slow [36] m-sequence (length of 2^14^-1), i.e., a pseudo-random succession of 0 (no checkerboard pattern reversal) and 1 (pattern reversal) states, each lasted 2 frames (26.6 ms) resulting in an average reversal rate of 18.75 reversals per second (rps). Stimuli, i.e., dartboard-checkerboard patterns with a mean luminance of 56 cd/m^2^, were presented on a monochrome CRT-monitor (MDG403, Philips; P45 phosphor) at a frame rate of 75 Hz, while the measurement was checked on a separate control monitor. The signals were amplified by 100 K (Grass Model 12, Astro-Med, Inc., West Warwick, RI, USA), band-pass filtered 3–300 Hz and digitized at 1200 Hz.

The mfPERGs were recorded binocularly with non-dilated pupils using DTL electrodes [37] placed in the upper margin of lower lid. The reference and ground cup electrodes filled with conductive paste (Ten20, WEAVER and Company, Aurora, CO, USA) were attached to the ipsilateral temple and forehead, respectively, after skin cleaning with a paste (skinPure, NIHON KODEN Corporation, Tokyo, Japan) to reduce the resistance of the skin below 5 kOhm. Refractive correction was optimized for a 36 cm viewing distances.

Participants underwent 3 repetitions of mfPERG recordings; each repetition divided was into 32 segments for patient comfort and optimization of recording quality. Signals in each segment that were contaminated with blinks or noise were discarded and repeated. The 1st-slice of the second order kernel were extracted using VERIS 5.1.12XScience. The polarity of the 2nd order kernels is, by convention, flipped with respect to the conventional recording and therefore, flipped back for offline analysis. Subsequent analyses were performed using Igor (IGOR Pro, WaveMetrics, Portland). Traces were digitally filtered (high pass filter: 3 Hz low pass filter: 45 Hz). Traces from right eyes were left-right flipped to match stimulated visual fields of traces recorded from left eyes of other participants.

From the mfPERG traces, we determined the amplitudes of the first negative trough from baseline; i.e., N1, the major positive peak, i.e., P1, from the trough of the preceding N1wave and the second negative trough i.e., N2, from the preceding P1 peak. N1, P1 and N2 are analogous to the transient PERG waves, i.e., N35, P50 and N95, respectively [10].

### 4.3. Optical Coherence Tomography (OCT)

OCT scans (Figure 4) were performed using a spectral domain OCT device (Heidelberg Spectralis^®^, Heidelberg Engineering, Heidelberg, Germany). Peripapillary retinal nerve fiber layer thickness (pRNFL) from a 3.5 mm circle scan centered on the optic disc (12° diameter) with 768 A-scan was acquired to calculate the averaged (pRNFL G), papillomacular bundle (pRNFL PMB) and temporal (pRNFL T) sectors thickness. The macula scan consisted of a custom-made scan comprising 61 vertical B-scans (each with 768 A-Scans, automatic real-time = 13 frames) with a scanning angle of 30° × 25° focusing on the fovea. Based on the macular scan, ganglion cell layer (GCL) thickness was computed using a beta software provided by Heidelberg Engineering that used a multilayer segmentation algorithm. Further, the outer retinal layer (ORL), outer nuclear layer (ONL), inner nuclear layer (INL), inner plexiform layer (IPL), retinal nerve fiber layer (RNFL) thickness were exploratively analyzed. Each layer thickness was averaged within the three rings of the nine ETDRS areas established by the Early Treatment Diabetic Retinopathy study (Figure 4), i.e., 1 mm (foveal), 3 mm (parafoveal), and 6 mm (perifoveal) rings.

### 4.4. Analysis and Statistics

The mfPERG recordings were analyzed using Igor (IGOR Pro, WaveMetrics, Portland). Statistical tests were conducted using R [38]. Normality test was conducted using Shapiro–Wilk test. Comparison of functional and structural measures were performed with an ANOVA except for mfPERG peak times (non-normally distributed data) tested with Kruskal–Wallis tests. Independent t-tests and Mann-Whitney tests were conducted the changes of each MS group vs. the healthy group for normally and non-normally distributed data, respectively. The association between structure and function indexes were evaluated using Pearson correlation. Holm Bonferroni adjust [39] was applied to correct for multiple testing for ANOVAs, Kruskal–Wallis, *t*-tests, Mann-Whitney tests and correlation tests.

## Figures and Tables

**Table 1:** Demographic and clinical characteristics of the participants.

	Control (N = 14)	NON (N = 12)	HON (N = 11)	*p*-Value
Age (y)	41.0 [12.6] (20–60)	42.0 [10.9] (27–61)	39.0 [9.8] (25–52)	0.803
Female N (%)	7 [50]	8 [66.7]	8 [72.7]	0.384
Disease duration (y)	-	6.4 [4.6] (1–13)	10.3 [8.0] (1–26)	0.179
BCVA (logMAR)	−0.02 (−0.1–0.00)	−0.02 (−0.1–0.1)	0.00 (−0.1–0.1)	0.54
Median EDSS	-	2.0 (1–7)	1.5 (0–4.5)	0.332

Unless otherwise reported mean [standard deviation] (range) is given; BCVA = best corrected visual acuity (logMAR = logarithm of minimum angle of resolution). EDSS = expanded disability status scale, HON = multiple sclerosis with a history of optic neuritis, N = number of subjects, NON = multiple sclerosis without a history of optic neuritis. Y = years. Disease duration was defined as timespan between symptom onset and visual measurements. Groups were compared with respect to categorical (using a c^2^-test) and continuous variables (using analysis of variance (ANOVA) or Kruskal–Wallis test and a t-test or Mann–Whitney U test).

**Table 2:** Multifocal pattern electroretinogram ring analysis of amplitudes and peak times.

		**N1**		**P1**		**N2**	
**Amplitude (µV)**	**Group**	**Mean [SD]**	**ANOVA F(2,34)**	**Mean [SD]**	**ANOVA F(2,34)**	**Mean [SD]**	**ANOVA F(2,34)**
Summed	Control	−5.36 [1.38]	1.2	11.96 [3.05]	0.8	−10.55 [2.48]	1.2
	NON	−5.05 [1.6]		11.46 [3.73]		−9.82 [3.26]	
	HON	−4.5 [1.08]		10.44 [2.29]		−8.86 [2.16]	
Ring 1	Control	−0.5 [0.16]	2	1.24 [0.37]	3.6	−1.5 [0.39]	7.8 **
	NON	−0.41 [0.11]		1.10 [0.24]		−1.16 [0.36]	
	HON	−0.4 [0.16]		0.92 [0.23]		−0.97 [0.24]	
Ring 2	Control	−1.05 [0.28]	1.3	−2.06 [0.44]	2.4	−2.06 [0.44]	4
	NON	−1.01 [0.38]		−1.77 [0.64]		−1.77 [0.64]	
	HON	−0.86 [0.24]		−1.51 [0.33]		−1.51 [0.33]	
Ring 3	Control	−1.84 [0.57]	2.3	4.09 [1.0]	1.4	−3.4 [0.68]	2.2
	NON	−1.69 [0.53]		3.89 [1.34]		−3.21 [1.15]	
	HON	−1.40 [0.41]		3.4 [0.67]		−2.7 [0.59]	
Ring 4	Control	−2.02 [0.63]	0.2	5.02 [1.49]	0.2	−4.52 [1.24]	0.3
	NON	−2.00 [0.69]		4.94 [1.58]		−4.35 [1.3]	
	HON	−1.88 [0.53]		4.62 [1.27]		−4.1 [1.29]	
**Peak Time (ms)**	**Group**	**Median (Range)**	**KW**	**Median (Range)**	**KW**	**Median (Range)**	**KW**
Summed	Control	25 (5.0)	4.9	45.42 (8.33)	5	73.33 (9.17)	0.1
	NON	25 (7.5)		43.33 (13.33)		72.92 (10.0)	
	HON	24.17 (4.17)		42.5 (6.67)		73.33 (6.67)	
Ring 1	Control	27.08 (10.83)	5.3	54.58 (11.67)	3.2	75.83 (18.33)	0.4
	NON	25.83 (16.67)		53.33 (19.17)		75.42 (15.83)	
	HON	25.83 (9.17)		52.5 (13.33)		76.67 (16.67)	
Ring 2	Control	25.83 (6.67)	7.8	49.58 (10.0)	10.2 *	73.33 (19.17)	0.7
	NON	25.42 (14.17)		45.83 (15.83)		72.92 (10.0)	
	HON	24.17 (4.17)		43.33 (11.67)		72.5 (20.0)	
Ring 3	Control	25 (5.0)	5.9	44.58 (8.33)	6.6	72.92 (15.83)	0.4
	NON	24.58 (7.5)		43.33 (11.67)		71.67 (15.0)	
	HON	23.33 (5.0)		41.67 (8.33)		73.33 (22.5)	
Ring 4	Control	25 (5.0)	3	43.33 (9.17)	2.4	70.42 (20.83)	1.4
	NON	24.17 (5.0)		42.5 (9.17)		70.83 (14.17)	
	HON	24.17 (4.17)		41.67 (7.5)		72.5 (11.67)	

N1 = first negative wave of multifocal pattern electroretinogram (mfPERG). P1 = positive wave of mfPERG. N2 = second negative wave of mfPERG. [SD] = Standard deviation. NON = multiple sclerosis without a history of optic neuritis; HON: multiple sclerosis with a history of optic neuritis. Groups were compared with respect to continuous variables (using analysis of variance (ANOVA) or Kruskal–Wallis test (KW) and a t-test, or Mann–Whitney U test). * *p* ≤ 0.05, ** *p* ≤ 0.01.

**Table 3:** Outer and inner retinal layers thickness.

**Macula**		**Center**		**Parafoveal**		**Perifoveal**	
**Thickness (µm)**	**Group**	**Mean [SD]**	**ANOVA F(2,34)**	**Mean [SD]**	**ANOVA F(2,34)**	**Mean [SD]**	**ANOVA F(2,34)**
Total	Control	279.71 [25.63]	0.2	344.43 [13.55]	3.8	298.89 [9.66]	1.4
	NON	282.67 [27.77]		337.48 [18.45]		293.52 [13.32]	
	HON	286.73 [28.63]		326.32 [17.1]		290.75 [13.94]	
mRNFL	Control	12.79 [2.75]	0.3	21.95 [1.58]	0.1	36.14 [2.65]	3
	NON	12.33 [3.06]		22.04 [2.39]		36.54 [5.9]	
	HON	13.18 [2.79]		21.64 [2.13]		32.05 [5.86]	
GCL	Control	16.00 [5.11]	0.2	51.75 [3.7]	13.8 ***	35.16 [2.8]	6.5 **
	NON	17.00 [8.5]		48.21 [6.2]		33.04 [2.9]	
	HON	18.09 [11.17]		40.14 [6.7]		30.73 [3.5]	
IPL	Control	21.43 [4.3]	0.2	41.84 [2.3]	8.2 **	28.88 [1.9]	3
	NON	22.92 [6.9]		39.46 [4.3]		27.38 [2.8]	
	HON	22.73 [6.4]		36.16 [3.8]		26.55 [2.6]	
	Control	18.71 [4.63]	0.2	46.79 [2.9]	12 ***	32.02 [2.32]	4.9 *
GCIPL	NON	19.96 [7.65]		43.83 [5.2]		30.21 [2.76]	
	HON	20.41 [8.72]		38.15 [5.04]		28.64 [3.02]	
INL	Control	20.29 [6.91]	1.3	40.82 [4.02]	0.4	32.52 [1.88]	0.4
	NON	21.42 [7.33]		39.6 [2.98]		32.06 [2.66]	
	HON	25.64 [10.85]		40.2 [2.4]		32.84 [1.94]	
ONL	Control	92.71 [8.84]	0.4	71.25 [5.83]	0.6	59.95 [5.37]	1.6
	NON	94.5 [15.22]		68.23 [8.43]		56.19 [5.74]	
	HON	89.18 [19.95]		70.0 [6.16]		58.25 [4.63]	
ORL	Control	90.86 [2.28]	0.5	81.77 [1.25]	2.3	78.21 [1.28]	2.7
	NON	91.17 [3.35]		83.38 [2.56]		79.69 [1.75]	
	HON	89.55 [6.01]		82.91 [2.0]		79.48 [2.28]	
**Optic disc**		**G**		**PMB**		**T**	
	**Group**	**Mean [SD]**	**F(2,32)**	**Mean [SD]**	**F(2,32)**	**Mean [SD]**	**F(2,32)**
pRNFL	Control	94.64 [5.84]	2.4	54.33 [11.77]	6.5 *	71.42 [14.89]	5.2 *
	NON	92.92 [11.41]		45.0 [6.81]		61.17 [10.97]	
	HON	86.18 [12.02]		40.82 [8.3]		55.18 [10.31]	

NON = multiple sclerosis without a history of optic neuritis; HON = multiple sclerosis with a history of optic neuritis; peripapillary retinal nerve fiber layer thickness: G = global average thickness, T = temporal, PMB = papillomacular bundle; mRNFL = macular retinal nerve fiber layer thickness; GCL = ganglion cell layer; IPL = inner plexiform layer; GCIPL = ganglion ell inner plexiform layer; INL = inner nuclear layer; ONL = Outer nuclear layer; ORL = Outer retinal layer; [SD] = standard deviation. Groups were compared with respect to continuous variables (using analysis of variance (ANOVA) and *t*-test).* *p* ≤ 0.05; ** *p* ≤ 0.01, *** *p* ≤ 0.001.

## Data Availability

Anonymized data will be shared by request from any qualified investigator.

## Supplementary Materials

The following are available online at https://www.mdpi.com/article/10.3390/ijms22073419/s1, Table S1: Correlation analysis of mfPERG vs. structural tests.

## References

[1] Yu J.G., Feng Y.F., Xiang Y., Huang J.H., Savini G., Parisi V., Yang W.J., Fu X.A. (2014). Retinal Nerve Fiber Layer Thickness Changes in Parkinson Disease: A Meta-Analysis. PLoS ONE.

[2] Alonso R., Gonzalez-Moron D., Garcea O. (2018). Optical coherence tomography as a biomarker of neurodegeneration in multiple sclerosis: A review. Mult. Scler. Relat. Disord..

[3] McDonald W.I., Barnes D. (1992). The ocular manifestations of multiple sclerosis. 1. Abnormalities of the afferent visual system. J. Neurol. Neurosurg. Psychiatry.

[4] Sriram P., Wang C., Yiannikas C., Garrick R., Barnett M., Parratt J., Graham S.L., Arvind H., Klistorner A. (2014). Relationship between Optical Coherence Tomography and Electrophysiology of the Visual Pathway in Non-Optic Neuritis Eyes of Multiple Sclerosis Patients. PLoS ONE.

[5] Petzold A., de Boer J.F., Schippling S., Vermersch P., Kardon R., Green A., Calabresi P.A., Polman C. (2010). Optical coherence tomography in multiple sclerosis: A systematic review and meta-analysis. Lancet Neurol..

[6] Saidha S., Syc S.B., Ibrahim M.A., Eckstein C., Warner C.V., Farrell S.K., Oakley J.D., Durbin M.K., Meyer S.A., Balcer L.J. (2011). Primary retinal pathology in multiple sclerosis as detected by optical coherence tomography. Brain.

[7] Wicki C.A., Hanson J.V.M., Schippling S. (2018). Optical coherence tomography as a means to characterize visual pathway involvement in multiple sclerosis. Curr. Opin. Neurol..

[8] Klistorner A., Sriram P., Vootakuru N., Wang C., Barnett M.H., Garrick R., Parratt J., Levin N., Raz N., Van der Walt A. (2014). Axonal loss of retinal neurons in multiple sclerosis associated with optic radiation lesions. Neurology.

[9] Holder G.E. (1997). The pattern electroretinogram in anterior visual pathway dysfunction and its relationship to the pattern visual evoked potential: A personal clinical review of 743 eyes. Eye.

[10] Bach M., Cuno A.-K., Hoffmann M.B. (2018). Retinal conduction speed analysis reveals different origins of the P50 and N95 components of the (multifocal) pattern electroretinogram. Exp. Eye Res..

[11] Berninger T.A., Heider W. (1990). Pattern electroretinograms in optic neuritis during the acute stage and after remission. Graefe’s Arch. Clin. Exp. Ophthalmol..

[12] Holder G.E. (1991). The incidence of abnormal pattern electroretinography in optic nerve demyelination. Electroencephalogr. Clin. Neurophysiol..

[13] Viswanathan S., Frishman L.J., Robson J.G. (2000). The uniform field and pattern ERG in macaques with experimental glaucoma: Removal of spiking activity. Investig. Ophthalmol. Vis. Sci..

[14] Wang J., Cheng H., Hu Y.-S., Tang R.A., Frishman L.J. (2012). The Photopic Negative Response of the Flash Electroretinogram in Multiple Sclerosis. Investig. Ophthalmol. Vis. Sci..

[15] Nakamura H., Miyamoto K., Yokota S., Ogino K., Yoshimura N. (2011). Focal macular photopic negative response in patients with optic neuritis. Eye.

[16] Hanson J.V.M., Hediger M., Manogaran P., Landau K., Hagenbuch N., Schippling S., Gerth-Kahlert C. (2018). Outer Retinal Dysfunction in the Absence of Structural Abnormalities in Multiple Sclerosis. Investig. Ophthalmol. Vis. Sci..

[17] Filgueiras T.G., Oyamada M.K., Preti R.C., Apóstolos-Pereira S.L., Callegaro D., Monteiro M.L.R. (2019). Outer Retinal Dysfunction on Multifocal Electroretinography May Help Differentiating Multiple Sclerosis From Neuromyelitis Optica Spectrum Disorder. Front. Neurol..

[18] Hokazono K., Raza A.S., Oyamada M.K., Hood D.C., Monteiro M.L.R. (2013). Pattern electroretinogram in neuromyelitis optica and multiple sclerosis with or without optic neuritis and its correlation with FD-OCT and perimetry. Doc. Ophthalmol. Adv. Ophthalmol..

[19] Shen T., You Y., Arunachalam S., Fontes A., Liu S., Gupta V., Parratt J., Wang C., Barnett M., Barton J. (2019). Differing Structural and Functional Patterns of Optic Nerve Damage in Multiple Sclerosis and Neuromyelitis Optica Spectrum Disorder. Ophthalmology.

[20] Gundogan F.C., Demirkaya S., Sobaci G. (2007). Is Optical Coherence Tomography Really a New Biomarker Candidate in Multiple Sclerosis?—A Structural and Functional Evaluation. Investig. Ophthalmol. Vis. Sci..

[21] Wilkins L.W. (2018). Progressive inner nuclear layer dysfunction in non-optic neuritis eyes in MS. Neurol.—Neuroimmunol. Neuroinflamm..

[22] Beykin G., Norcia A.M., Srinivasan V.J., Dubra A., Goldberg J.L. (2021). Discovery and clinical translation of novel glaucoma biomarkers. Prog. Retin. Eye Res..

[23] Pawlitzki M., Horbrügger M., Loewe K., Kaufmann J., Opfer R., Wagner M., Al-Nosairy K.O., Meuth S.G., Hoffmann M.B., Schippling S. (2020). MS optic neuritis-induced long-term structural changes within the visual pathway. Neurol. Neuroimmunol. Neuroinflamm..

[24] Scheel M., Finke C., Oberwahrenbrock T., Freing A., Pech L.-M., Schlichting J., Sömmer C., Wuerfel J., Paul F., Brandt A.U. (2014). Retinal nerve fibre layer thickness correlates with brain white matter damage in multiple sclerosis: A combined optical coherence tomography and diffusion tensor imaging study. Mult. Scler. (Houndmills Basingstoke Engl.).

[25] Klistorner A., Arvind H., Nguyen T., Garrick R., Paine M., Graham S., Yiannikas C. (2009). Fellow eye changes in optic neuritis correlate with the risk of multiple sclerosis. Mult. Scler. (Houndmills Basingstoke Engl.).

[26] Klistorner A., Graham E.C., Yiannikas C., Barnett M., Parratt J., Garrick R., Wang C., You Y., Graham S.L. (2017). Progression of retinal ganglion cell loss in multiple sclerosis is associated with new lesions in the optic radiations. Eur. J. Neurol..

[27] Oberwahrenbrock T., Ringelstein M., Jentschke S., Deuschle K., Klumbies K., Bellmann-Strobl J., Harmel J., Ruprecht K., Schippling S., Hartung H.-P. (2013). Retinal ganglion cell and inner plexiform layer thinning in clinically isolated syndrome. Mult. Scler. (Houndmills Basingstoke Engl.).

[28] Petzold A., Balcer L.J., Calabresi P.A., Costello F., Frohman T.C., Frohman E.M., Martinez-Hernandez E., Green A.J., Kardon R., Outteryck O. (2017). Retinal layer segmentation in multiple sclerosis: A systematic review and meta-analysis. Lancet Neurol..

[29] Porciatti V., Ventura L.M. (2009). Physiological significance of steady-state PERG losses in glaucoma: Clues from simulation of abnormalities in normal subjects. J. Glaucoma.

[30] Kreuz A.C., de Moraes C.G., Hatanaka M., Oyamada M.K., Monteiro M.L.R. (2018). Macular and Multifocal PERG and FD-OCT in Preperimetric and Hemifield Loss Glaucoma. J. Glaucoma.

[31] Ganekal S., Dorairaj S., Jhanji V. (2013). Pattern Electroretinography Changes in Patients with Established or Suspected Primary Open Angle Glaucoma. J. Curr. Glaucoma Pract..

[32] Hood D.C., Frishman L.J., Saszik S., Viswanathan S. (2002). Retinal Origins of the Primate Multifocal ERG: Implications for the Human Response. Investig. Ophthalmol. Vis. Sci..

[33] Ikeda H., Franchi A., Turner G., Shilling J., Graham E. (1989). Electroretinography and electro-oculography to localize abnormalities in early-stage inflammatory eye disease. Doc. Ophthalmol..

[34] Polman C.H., Reingold S.C., Banwell B., Clanet M., Cohen J.A., Filippi M., Fujihara K., Havrdova E., Hutchinson M., Kappos L. (2011). Diagnostic criteria for multiple sclerosis: 2010 revisions to the McDonald criteria. Ann. Neurol..

[35] Kurtzke J.F. (1983). Rating neurologic impairment in multiple sclerosis: An expanded disability status scale (EDSS). Neurology.

[36] Hoffmann M.B., Flechner J.-J. (2008). Slow pattern-reversal stimulation facilitates the assessment of retinal function with multifocal recordings. Clin. Neurophysiol..

[37] Dawson W.W., Trick G.L., Litzkow C.A. (1979). Improved electrode for electroretinography. Investig. Ophthalmol. Vis. Sci..

[38] R Core Team (2013). R: The R Project for Statistical Computing. R: A Language and Environment for Statistical Computing.

[39] Holm S. (1979). A Simple Sequentially Rejective Multiple Test Procedure. Scand. J. Stat..

