# Supplementary material for: Structure–Function Relationship of Retinal Ganglion Cells in Multiple Sclerosis
Source: Int J Mol Sci. 2021 Mar 26;22(7):3419. doi: 10.3390/ijms22073419 (PMC8037992; doi:10.3390/ijms22073419)
Supplement: Supplementary file 1 [file ijms-22-03419-s001.pdf]

Table S1. Correlation analysis of mfPERG vs structural tests.

	**N1 Amplitude [μV]** r Pearson correlation, P unadjusted			
Thickness [μm]	Ring1	Ring 2	Ring 3	Ring 4
cORL	0.37, 0.024	0.34, 0.037	0.27, 0.11	0.14, 0.42
pfORL	0.28, 0.09	0.32, 0.057	0.36, 0.03	0.17, 0.32
pOR	0.19, 0.25	0.19, 0.25	0.29, 0.08	0.15, 0.39
cONL	-0.18, 0.28	-0.21, 0.20	-0.12, 0.49	-0.01, 0.96
pfONL	-0.16, 0.33	-0.27, 0.10	-0.11, 0.50	0.003, 0.99
pONL	-0.14, 0.40	-0.27, 0.10	-0.12, 0.49	0.02, 0.89
	**P1 Amplitude [μV]** r Pearson correlation, P unadjusted			
Thickness [μm]	Ring1	Ring 2	Ring 3	Ring 4
cORL	-0.16, 0.36	-0.25, 0.13	-0.29, 0.08	-0.05, 0.76
pfORL	-0.29, 0.08	-0.38, 0.02	-0.39, 0.018	-0.17, 0.32
pOR	-0.28, 0.09	-0.26, 0.11	-0.30, 0.07	-0.15, 0.38
cONL	0.03, 0.88	0.15, 0.38	0.13, 0.45	0.07, 0.67
pfONL	0.13, 0.45	0.20, 0.24	0.14, 0.40	0.02, 0.92
pONL	0.19, 0.26	0.20, 0.22	0.11, 0.51	-0.03, 0.87
cINL	0.06, 0.72	-0.05, 0. 57	-0.15, 0.39	-0.22, 0.19
pfINL	0.36, 0.03	0.30, 0.07	0.07, 0.68	0.06, 0.74
pINL	0.35, 0.036	0.31, 0.06	0.37, 0.026	0.40, 0.015
	**N2 Amplitude [μV]** r Pearson correlation, P unadjusted			
Thickness [μm]	Ring1	Ring 2	Ring 3	Ring 4
pRNFL G	-0.26, 0.12	-0.16, 0.34	-0.24, 0.16	-0.36, 0.026
pRNFL PMB	-0.33, 0.051	-0.13, 0.46	0.001, 0.99	0.18, 0.30
pRNFL T	-0.31, 0.07	-0.12, 0.50	-0.05, 0.77	0.08, 0.67
cINL	0.13, 0.43	0.17, 0.32	0.26, 0.12	0.27, 0.11
pfINL	-0.12, 0.47	-0.17, 0.30	-0.02, 0.86	-0.01, 0.95
pINL	-0.32, 0.051	-0.38, 0.02	-0.38, 0.02	-0.42, 0.01
cIPL	0.11, 0.53	0.12, 0,48	0.16, 0.34	0.26, 0.12
pfIPL	-0.25, 0.14	-0.08, 0.63	0.013, 0.94	0.04, 0.83
pIPL	-0.39, 0.017	-0.20, 0.23	-0.17, 0.31	-0.24, 0.15
cGCL	0.14, 0.42	0.18, 0.29	0.23, 0.18	0.33, 0.045
pfGCL	-0.28, 0.09	-0.11, 0.54	-0.07, 0.67	0.07, 0.68
pGCL	-0.47, 0.003	-0.28, 0.09	-0.29, 0.08	-0.27, 0.11
cGCIPL	0.13, 0.46	0.16, 0.36	0.20, 0.23	0.33, 0.07
pfGCIPL	-0.27, 0.10	-0.10, 0.56	-0.04, 0.81	0.06, 0.73
pGCIPL	-0.45, 0.005	-0.25, 0.13	-0.25, 0.14	-0.26, 0.12
cMRNFL	0.08, 0.62	0.15, 0.39	0.28, 0.09	0.27, 0.11
pfMRNFL	0.11, 0.53	0.16, 0.33	0.25, 0.14	0.18, 0.29
pMNFL	-0.14, 0.41	-0.06, 0.74	-0.06, 0.71	-0.07, 0.67

mfPERG amplitudes are converted to positive values.
pRNFL= average peripapillary retinal nerve fiber layer thickness:
G: averaged, PMB: papillomacular bundle, T: Temporal sector.
A suffix of c, pf, and p: central, parafoveal and perifoveal rings of ETDRS macular scans; ORL: Outer retinal layer; ONL= Outer nuclear layer;
INL: Inner nuclear layer; IPL: Inner plexiform layer; GCL: Ganglion cell layer;
GCIPL; ganglion cell inner plexiform layer;
MRNFL= macular retinal nerve fiber layer.
